# Increased *FGF19* copy number is frequently detected in hepatocellular carcinoma with a complete response after sorafenib treatment

**DOI:** 10.18632/oncotarget.10077

**Published:** 2016-06-15

**Authors:** Masaki Kaibori, Kazuko Sakai, Morihiko Ishizaki, Hideyuki Matsushima, Marco A. De Velasco, Kosuke Matsui, Hiroya Iida, Hiroaki Kitade, A-Hon Kwon, Hiroaki Nagano, Hiroshi Wada, Seiji Haji, Tadashi Tsukamoto, Akishige Kanazawa, Yutaka Takeda, Shigekazu Takemura, Shoji Kubo, Kazuto Nishio

**Affiliations:** ^1^ Department of Surgery, Hirakata Hospital, Kansai Medical University, Hirakata, Osaka, 573-1010, Japan; ^2^ Department of Genome Biology, Kinki University Faculty of Medicine, Osakasayama, Osaka, 589-8511, Japan; ^3^ Department of Gastroenterological Surgery, Osaka University Graduate School of Medicine, Osaka, 565-0871, Japan; ^4^ Department of Surgery, Kinki University Faculty of Medicine, Osakasayama, Osaka, 589-8511, Japan; ^5^ Department of Hepato-Biliary-Pancreatic Surgery, Osaka City General Hospital, Miyakojima, Osaka, 534-0024, Japan; ^6^ Department of Surgery, Kansai Rosai Hospital, Amagasaki, Hyogo, 660-8511, Japan; ^7^ Department of Hepato-Biliary-Pancreatic Surgery, Osaka City University Graduate School of Medicine, Osaka, 558-8585, Japan

**Keywords:** FGF19, sorafenib, copy number gain, hepatocellular carcinoma

## Abstract

The multi-kinase inhibitor sorafenib is clinically approved for the treatment of patients with advanced hepatocellular carcinoma (HCC). We previously reported that fibroblast growth factor 3 and 4 (*FGF3/FGF4*) amplification is a predictor of a response to sorafenib. This study aims to analyze the relationship between *FGF*-*FGF* receptor (*FGFR*) genetic alterations and the response to sorafenib. Formalin-fixed, paraffin-embedded tissue specimens from HCC patients who had achieved a complete response (CR, N=6) or non-CR (N=39) to sorafenib were collected and were examined for *FGF*-*FGFR* gene alterations using next generation sequencing and copy number assay. *FGFR* mutations were detected in 5 of 45 (11.1%) cases. There was no significant association between *FGFR* mutation status and the response to sorafenib. We detected no increase in the *FGF3/FGF4* copy number in CR cases. An *FGF19* copy number gain was detected more frequently among CR cases (2/6, 33.3%) than among non-CR cases (2/39, 5.1%) (*P* = 0.024, Chi-squared test). In conclusion, a copy number gain for *FGF19* may be a predictor of a response to sorafenib, in addition to *FGF3*/*FGF4* amplification.

## INTRODUCTION

Hepatocellular carcinoma (HCC) is the most common primary malignancy of the liver [1]. Several reports have suggested that hepatocarcinogenesis involves multiple molecular pathways involving the accumulation of genetic and epigenetic alterations, including copy number aberration and gene mutations [2] [3] [4]. Sorafenib is a multi-targeted kinase inhibitor that has a potent antitumor activity in several preclinical models [5]. Sorafenib is now used as standard therapy for advanced HCC [6] [7]. However, a complete response is very rare, and the response rate is low (between 0.7% and 3.3%) [6] [7]. Nevertheless, complete responses were observed in patients with advanced HCC after short-term treatment with sorafenib [8] [9] [10]. We previously reported the clinical and molecular backgrounds of 13 responders to sorafenib with significant tumor shrinkage in a retrospective study [11]. A comparative genomic hybridization analysis using one frozen HCC sample from a responder demonstrated that the 11q13 region, a rare amplicon in HCC including the loci for *FGF3* and *FGF4*, was highly amplified. However, the mechanisms responsible for the responses to sorafenib in the remaining cases without *FGF3/FGF4* amplification remain unclear.

In this study, we further analyze molecular alterations on the FGF-FGFR signal pathway using the different sample cohort from that of our previous report [11]. The FGF pathway is aberrantly activated through a variety of genetic alterations in many types of cancers [12]. Recently, several studies have reported that gene mutation or amplification in *FGF/FGFR* predict for sensitivity of FGFR inhibitors. *FGFR1, FGFR2* or *FGF19* gene amplifications were reported as potential biomarkers of a selective FGFR inhibitor [13] [14]. Thus, a genetic alteration in *FGF/FGFR* is considered to be a potential biomarker for effective FGFR inhibition. Here, we focused on *FGF/FGFR* gene alterations to elucidate other mechanisms related to sorafenib response because sorafenib potentially inhibits FGFR kinase activity [5] [15]. In the current study, we collected responder cases (PR and CR) to sorafenib and conducted a case-control study with a retrospective design in order to explore the association between the efficacy of sorafenib and gene alterations, including copy number changes and mutations, in FGF-FGFR signals.

## RESULTS

### Baseline characteristics

The characteristics of the patients are summarized in Table 1. The tumor samples of complete response (CR) (N=6) and partial response (PR) (N=4) cases were collected. Samples of stable disease (SD) (N=13) and progress disease (PD) (N=22) cases were also collected as control. Patients with recurrence after curative surgery were treated with sorafenib. The median time from surgery to initiation of sorafenib therapy of CR, PR, SD, and PD cases were 35.8 months (range, 9.8-82.1), 32.5 months (range, 20.4-57.8), 9.6 months (range, 1.4-66.3), and 1.4 months (range, 0.4-56.9), respectively. Three of the six CR cases (case no. CR4, CR5, and CR6) received transcatheter arterial chemoembolization (TACE) before starting sorafenib treatment. Two cases (case no. CR1 and CR2) received TACE before and during treatment with sorafenib. One case (case no. CR3) received TACE/hepatic arterial infusion chemotherapy (HAIC) before and during treatment with sorafenib. Three PR cases (case no. PR1, PR2, and PR4) received TACE, TACE/radiation, and TACE/radiofrequency ablation (RFA) before sorafenib treatment, respectively. One PR case (case no. PR3) received only sorafenib treatment. Ten of the 13 SD cases received TACE before starting sorafenib treatment. Eight of 22 PD cases received TACE before starting sorafenib treatment. The remaining 17 SD and PD cases received only sorafenib treatment. Median time from first drug administration to the date of the first clinical complete and partial response was 4.0 months (range; 1.5-33.7) and 2.2 months (range; 1.7-14.2), respectively.

**Table 1:** Patient characteristics (N=45)

	CR (N=6)	PR (N=4)	SD (N=13)	PD (N=22)
Age (years)				
Median (range)	76.5 (70–80)	71.0 (67-83)	67.0 (57-86)	68.0 (45-82)
Sex				
Male (%)	4 (66.7)	3 (75.0)	12 (92.3)	16 (72.7)
Female (%)	2 (33.3)	1 (25.0)	1 (7.7)	6 (27.3)
Hepatitis virus status				
HBV (%)	1 (16.7)	0 (0)	1 (7.7)	2 (9.1)
HCV (%)	2 (33.3)	2 (50.0)	6 (46.2)	9 (40.9)
HBV/HCV (%)	0 (0)	0 (0)	0 (0)	3 (13.6)
NBNC (%)	3 (50.0)	2 (50.0)	6 (46.2)	8 (36.4)
Child-Plug class				
A (%)	6 (100)	4 (100)	12 (92.3)	21 (95.5)
B (%)	0 (0)	0 (0)	1 (7.7)	1 (4.5)
Tumor size (cm)				
Median (range)	3.7 (2.0–7.0)	2.9 (2.5-4.5)	7.0 (1.5-25.0)	9.0 (1.4-65.0)
Number of tumors				
Single (%)	6 (100)	3 (75.0)	9 (69.2)	12 (54.5)
Multiple (%)	0 (0)	1 (25.0)	4 (30.8)	10 (45.5)
Histology				
Well differentiated (%)	1 (16.7)	0 (0)	2 (15.4)	1 (4.5)
Moderately differentiated (%)	5 (83.3)	2 (50.0)	11 (84.6)	18 (81.8)
Poorly differentiated (%)	0 (0)	2 (50.0)	0 (0)	3 (13.6)
TNM stage				
I (%)	4 (66.7)	3 (75.0)	1 (7.7)	1 (4.5)
II (%)	2 (33.3)	0 (0)	8 (61.5)	8 (36.4)
III (%)	0 (0)	1 (25.0)	2 (15.4)	11 (50.0)
IV (%)	0 (0)	0 (0)	2 (15.4)	2 (9.1)

Abbreviations: CR, complete response; PR, partial response; SD, stable disease; PD, progress disease; HBV, hepatitis B; HCV, hepatitis C; NBNC, non-hepatitis B and –C.

The majority of the cases exhibited Child-Plug class A, single tumor and moderate differentiation. Advanced stages (TNM stages III and IV) were classified in non-responder (SD and PD) cases.

### *FGFR* mutation analysis in complete or partial responders

Genomic DNA was subjected to *FGFR1-4* mutation analysis. We screened all exons of FGFRs (*FGFR1*, *FGFR2*, *FGFR3*, and *FGFR4*) in 45 cases. We identified *FGFR2* mutations in 2 (4.4%), *FGFR3* mutations in 2 (4.4%), and *FGFR1* and *FGFR4* mutations in 1 each (2.2%) (Supplementary Table S1). One CR case exhibited an *FGFR1^S602F^* mutation located in the kinase domain. Another PR case exhibited both *FGFR2^M538I^* and *FGFR4^G665E^* mutations, also located in the kinase domain. No mutations were found in the remaining eight cases. *FGFR3* mutations were detected only in SD or PD cases. In liver cancer, no mutation of the *FGFR1* gene was found on the TCGA database (cBioPortal database). *FGFR1^S602F^* was reported in only one case of melanomaand *FGFR2*, and *FGFR4* mutations were found in 4/231 (1.7%) and 1/231 (0.4%) liver cancer samples, respectively. Neither *FGFR2^M538I^* nor *FGFR4^G665E^* mutations were found in any of the studies available on the cBioPortal database. No experimental reports have discussed the functional changes associated with *FGFR1^S602F^*, *FGFR2^M538I^*, or *FGFR4^G665E^* mutations. We retrieved functional impact scores from the Mutation Assessor database. A higher score of predicted functional impact indicates a higher likelihood of a functional mutation (i.e., a driver mutation). A low predicted functional impact of the *FGFR1^S602F^* mutation was obtained. On the other hand, *FGFR2^M538I^* or *FGFR4^G665E^* mutations were projected to produce a medium functional impact. We analyzed the association between *FGFR* mutation status with sorafenib response and the following variables: sex, virus infection, Child-Plug class, tumor size, and number of tumors. As shown in Table 2, there was no significant association between mutation status and the clinical variables.

**Table 2:** Associations of *FGF19* copy number alterations, FGFR mutations, and clinical variables

	N	*FGF19* copy number gain		*P*	*FGFR* mutation^a)^		*P*
		Negative cases No. (%)	Positive cases No. (%)		Negative cases No. (%)	Positive cases No. (%)	
Response to sorafenib (RECIST ver1.1)							
CR	6	4 (66.7)	2 (33.3)		5 (83.3)	1 (16.7)	
Non-CR (PR, SD, PD)	39	37 (94.9)	2 (5.1)	0.024*	35 (89.7)	4 (10.3)	0.642
Sex							
Male (%)	35	32 (91.4)	3 (8.6)		31 (88.6)	4 (11.4)	
Female (%)	10	9 (90.0)	1 (10.0)	0.889	9 (90.0)	1 (10.0)	0.899
Hepatitis virus status							
HBV or HCV positive (%)	26	23 (88.5)	3 (11.5)		23 (88.5)	3 (11.5)	
NBNC (%)	19	18 (94.7)	1 (5.3)	0.465	17 (89.5)	2 (10.5)	0.915
Child-Plug class							
A (%)	43	39 (90.7)	4 (9.3)		38 (88.4)	5 (11.6)	
B (%)	2	2 (100.0)	0 (0.0)	0.651	2 (100.0)	0 (0.0)	0.609
Tumor size (cm)							
Median (range)		7.0 (1.1-65.0)	3.5 (2.8-6.0)	0.123	5.8 (1.4-65.0)	9.0 (2.5-11.0)	0.594
Number of tumors							
Single (%)	30	27 (90.0)	3 (10.0)		26 (86.7)	4 (13.3)	
Multiple (%)	15	14 (93.3)	1 (6.7)	0.711	14 (93.3)	1 (6.7)	0.502
Histology							
Well differentiated (%)	4	4 (100.0)	0 (0.0)		3 (75.0)	1 (25.0)	
Moderately differentiated (%)	36	33 (91.7)	3 (8.3)		34 (94.4)	2 (5.6)	
Poorly differentiated (%)	5	4 (80.0)	1 (20.0)	ND	3 (60.0)	2 (40.0)	ND
TNM stage							
I (%)	9	7 (77.8)	2 (22.2)		8 (88.9)	1 (11.1)	
II (%)	18	16 (88.9)	2 (11.1)		15 (83.3)	3 (16.7)	
III (%)	14	14 (100.0)	0 (0.0)		13 (92.9)	1 (7.1)	
IV (%)	4	4 (100.0)	0 (0.0)	ND	4 (100.0)	0 (0.0)	ND

Abbreviations: CR, complete response; PR, partial response; SD, stable disease; PD, progress disease; ND, not determined.

* *P* < 0.05 (Chi-squared test)

a Mutations in *FGFR1*, *FGFR2*, *FGFR3*, and *FGFR4*

### Copy number alterations in *FGF* and *FGFR* genes

**Figure 1:**
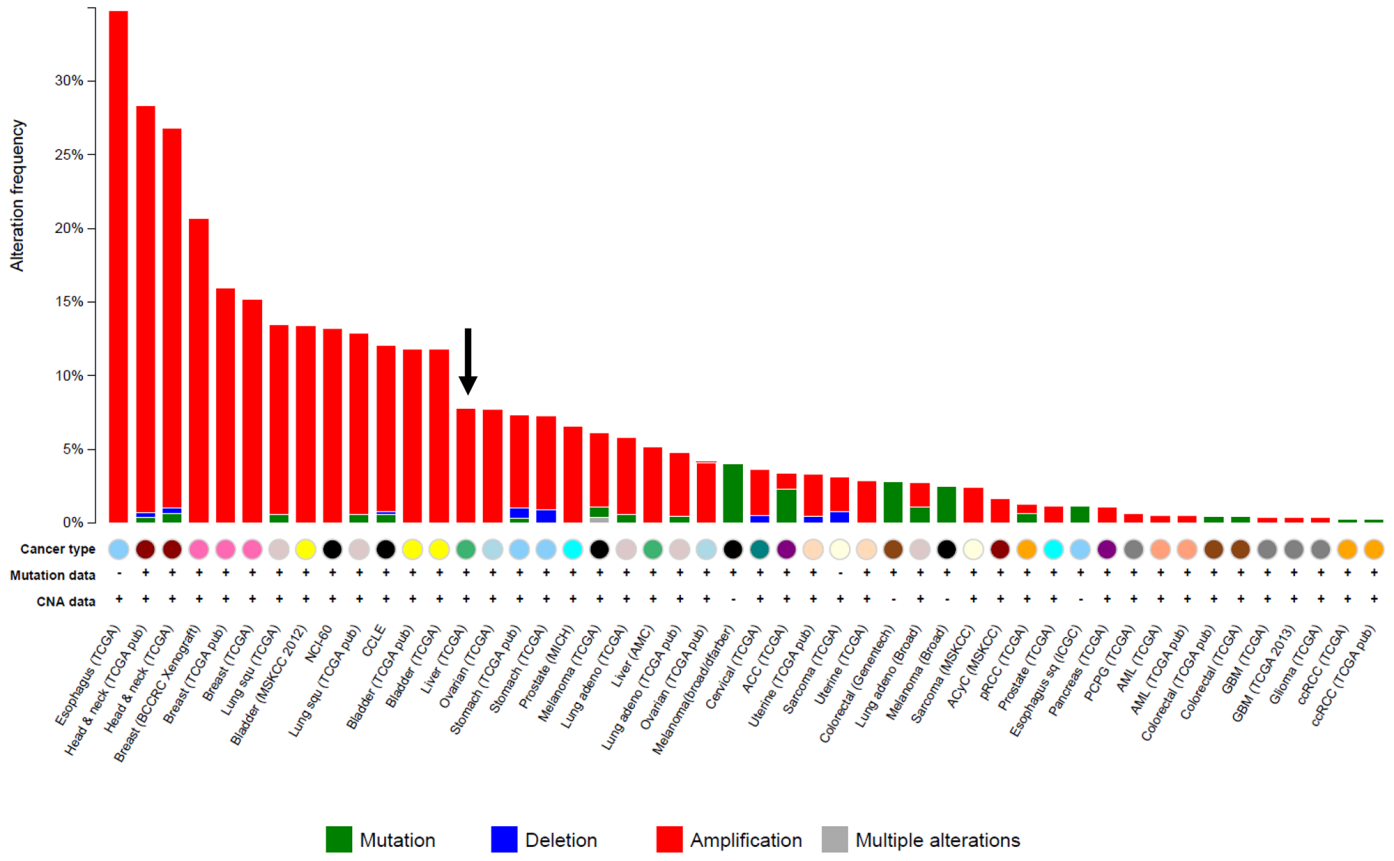
Frequency of *FGF19* gene amplification in solid cancers Gene mutation and copy number alterations in the *FGF19* gene were sought using the cBioPortal for Cancer Genomics (http://www.cbioportal.org/public-portal/). The arrow indicates the amplification of *FGF19* in HCC.

Copy number alterations in the tumor samples were analyzed using TaqMan chemistry. We screened the copy numbers of *FGF3, FGF4*, *FGF19, FGFR1, FGFR2, FGFR3,* and *FGFR4* in CR and PR samples. In this assay, a cut-off value for copy number gain was set at 5.00 for each gene. Increased copy numbers of *FGF19* were detected in two CR (2/6, 33.3%) cases. No copy number gains were detected for *FGF3*, *FGF4*, *FGFR1, FGFR2, FGFR3,* or *FGFR4*. To investigate whether an *FGF19* copy number gain was associated with a response to sorafenib, we analyzed additional tumor specimens from 35 patients who did not respond to sorafenib. Thirteen patients had SD and 22 had PD, as evaluated after sorafenib treatment. Increased copy numbers of *FGF19* were detected in 2/35 (5.7%) cases: 1/13 (7.7%) among SD cases and 1/22 (4.5%) among PD cases. *FGF3/FGF4* copy numbers were concomitantly increased with *FGF19* in one of each case. No copy number gains were detected for *FGFR1, FGFR2, FGFR3* or *FGFR4.* Additionally, increased *FGF19* copy numbers and *FGFR* mutations were mutually exclusive. We compared the frequency of *FGF19* copy number gain in these cases. An *FGF19* copy number gain was detected more frequently among CR cases than among non-CR cases (*P* = 0.024, Chi-squared test) (Table 2 and Supplementary Table S1). As shown in Table 2, there was no significant relationship between increased *FGF19* copy number and sex, virus infection, Child-Plug class, tumor size, and number of tumors. We also confirmed the frequency of *FGF19* copy number alterations using cBioPortal for Cancer Genomics (Figure 1). The frequency of *FGF19* amplification in a TCGA liver cancer dataset (15/193, 7.8%) was consistent with the data obtained from the 35 non-responders. *FGF19* gene amplification was considered to be a possible predictive biomarker for the efficacy of sorafenib in patients with HCC.

## DISCUSSION

In this report, we analyzed a set of patients treated with sorafenib after recurrence to curative surgery and found an increase in *FGF19* copy number alterations in pretreatment tissues from CR cases compared to those from non-CR. The median time from surgery to initiation of sorafenib therapy of CR/PR cases was longer than that of SD/PD cases. Although this result might suggest that duration from surgery to the initiation of sorafenib treatment is related with response to sorafenib, this association might be confounded by the time to recurrence.

Sorafenib, which has been clinically approved for the treatment of patients with advanced HCC [6] [7], is a multi-targeted kinase inhibitor [5]. The genetic alteration in *FGFR* has been frequently identified as an actionable biomarker [14] [16]. FGFR is not a major target for sorafenib, however, the off-target effect of sorafenib on FGFR might be clinically relevant for HCC tumors with FGF-FGFR alterations. We previously reported that *FGF3/FGF4* amplification in HCC might be involved in the response of tumors to sorafenib treatment. However the frequency of *FGF3/FGF4* amplification was 1-2% in Japanese HCC cases [11]. In the present study, we found no increases in the copy number of the responders (CR and PR) compared to that of non-responders (SD and PD), however, this was a different sample cohort from that of our previous report [11]. It is possible that differences between the backgrounds of these two cohorts that may have contributed to variances, however, this remains unclear. Potential confounding factors may be attributable to the following: 1) Sample size of this cohort was too small to conclude no amplification was detected in responder cases (0/10); 2) The criteria for responders in current cohort was different from that of the previous one; in this study a “responder” means CR+PR, whereas a “hyper-responder” means CR. Therefore, we investigated the associations between tumor genetic alterations in FGF-FGFR signals and the efficacy of sorafenib in patients with HCC.

A copy number assay revealed that *FGF19* amplification was observed in 4/45 (8.9%) cases. *FGF19* gene amplification has been reported in 7.8% of liver cancers in the TCGA database (cBioPortal database), consistent with the frequency observed in the present study. In our sample set, the frequency of *FGF19* amplification among the CR cases was significantly higher than that among the other cases (PR + SD + PD). FGF19 signaling is mediated via FGFR4, and FGF19–FGFR4 signaling is implicated in hepatocellular tumorigenesis [17] [18]. Molecular studies looking at the role of specific amplicons in HCC identified *FGF19* amplification as a potential driver gene of HCC [18]. Preclinical studies showed that the tumors with *FGF19* amplification are reportedly associated with a cellular sensitivity to FGFR inhibitors [14]. In the current study, it remains unclear whether increased *FGF19* copy numbers correlate with mRNA and protein expression. However, previous reports showed that *FGF19* copy number gain correlated well with levels of RNA and protein expression in HCC [18] [19]. Consequently, we speculated that a copy number gain of *FGF19* might be a predictor of a response to sorafenib, in addition to *FGF3*/*FGF4* amplification.

Next-generation sequencing enables a comprehensive analysis of genomic alterations. We focused on FGFR signals and performed targeted DNA sequencing for *FGFR1*, *FGFR2*, *FGFR3*, and *FGFR4*. In this study, 11.1% (5/45) of the HCC tumors had *FGFR* mutations, which is slightly higher than the previous report [20]. The frequency of *FGFR* mutation was not correlated with any clinicopathologic parameters including response to sorafenib. Therefore, we could not establish the significance of the *FGFR* mutation status and sensitivity to sorafenib. In addition, the biological significance of each mutation remains unclear thus we will need to analyze the functional meaning of each mutation in a future study.

We utilized a retrospective case-control study to explore biomarkers associated with sorafenib response, however, the sample size posed some limitations and there is an inherent susceptibility of selection bias with this type of study design. Therefore, prospective studies will be needed to further evaluate *FGF19* copy number gain as a biomarker of response to sorafenib.

In summary, a copy number gain of *FGF19* may serve as a potential candidate marker that predicts response to sorafenib, in addition to *FGF3/FGF4* amplification. Clinical studies examining a biomarker-driven enriched subpopulation are necessary to determine the efficacy of molecular-targeted treatments with kinase inhibitors. Therefore, the present results suggest new biomarkers for the enrichment of responders to kinase inhibitors.

## MATERIALS AND METHODS

### Patients and samples

Tumor specimens were obtained from a total of 45 HCC patients who had undergone sorafenib treatment at six hospitals between 2004 and 2013. Patients with recurrence after curative surgery were treated with sorafenib. Patients received sorafenib at doses ranging from 100 – 800 mg/ day based on tolerability and drug-related toxicities [21]. Tumor responses were evaluated using the Response Evaluation Criteria In Solid Tumors (RECIST) ver 1.1 criteria [22]: a complete response (CR) was defined as the disappearance of all measurable and evaluable evidence of disease; a partial response (PR) was defined as a > 30% decrease in the sum of the longest diameters of target lesions; stable disease (SD) is defined as a less 30% decrease or less than 20% increase in the sum of longest diameters; and progressive disease (PD) was indicated by a >20% increase in the sum of the longest diameters of target lesions or the appearance of any new lesion. Sixteen of the 22 PD cases showed >20% increase in tumor size. Remaining six cases showed bone, lung, and adrenal metastasis. This study was approved by the ethics committee of each institution. All the patients enrolled in the study had provided written informed consent for the use of resected samples. Among the 45 patients, 6 obtained a CR, four had PR, 13 showed SD and 22 showed PD. The study was approved by our Institutional Review Board (Kinki University Faculty of Medicine IRB approval no 25-106) and conformed to the ethical guidelines of the Declaration of Helsinki.

### DNA extraction

All FFPE specimens underwent a histological review, and only those containing sufficient tumor cells (at least 70% tumor cells) as revealed using hematoxylin-eosin staining were subjected to DNA extraction. The DNA was purified using an Allprep DNA/RNA FFPE kit (Qiagen, Valencia, CA) according to the manufacturer's instructions. The quality and quantity of the DNA were verified using the NanoDrop 2000 device (Thermo Scientific, Wilmington, DE) and the PicoGreen dsDNA assay kit (Life Technologies, Foster City, CA). The extracted DNA was stored at −80°C until analysis.

### DNA sequencing

Ten nanograms of DNA were used for the multiplex PCR amplification using the Ion AmpliSeq Library kit 2.0 (Life Technologies). We used a custom panel designed for *FGFR1*, *FGFR2*, *FGFR3*, and *FGFR4* genes. The Ion Xpress Barcode Adapters (Life Technologies) were ligated into the PCR products and were purified with Agencourt AMPure XP beads (Beckman Coulter, Brea, CA). The purified libraries were then pooled and sequenced using an Ion Torrent PGM device (Life Technologies) using the Ion PGM 200 Sequencing kit v2 (Life Technologies) and the Ion 318 v2 Chip kit (Life Technologies). DNA sequencing data were accessed through the Torrent Suite v.4.0 software program. Reads were aligned against the hg19 human reference genome, and variants were called using the variant caller ver. 4.0. Raw variant calls were filtered out using the following annotations: homozygous and heterozygous variants, quality score of <100 and depth of coverage <19. Germline mutations were excluded using the Human Genetic Variation Database (http://www.genome.med.kyoto-u.ac.jp/SnpDB) [23].

### Copy number assay

The copy numbers for *FGF3*, *FGF4*, *FGF19*, *FGFR1*, *FGFR2*, *FGFR3*, and *FGFR4* were determined using commercially available and predesigned TaqMan Copy Number Assays according to the manufacturer's instructions (Applied Biosystems, Foster City, CA), as described previously [11]. FAM-labeled primers for the target gene and a VIC-labeled primer for *TERT* as an endogenous control were used for the duplex assay. The primer IDs used for the FGFRs and FGFs were as follows: *FGF3*, Hs06336027_cn; *FGF4*, Hs01235235_cn; *FGF19*, Hs00147838_cn; *FGFR1*, Hs02164585_cn; *FGFR2*, Hs05208783_cn; *FGFR3*, Hs00113109_cn; and *FGFR4*, Hs01949336_cn. The copy numbers of the target genes were determined by relative quantification using the Copy-Caller-Software, v1.0 (Applied Biosystems). Normal female genomic DNA (Promega; Madison, WI) was used as the normal control (two copies). A cut-off value for copy number gain was set at 5.00 for each gene as described previously [11].

### Bioinformatics analysis of genomics of HCC

To analyze the prevalence of genomic alterations in the *FGF19* gene in HCC tissues, the database of the cBioPortal for Cancer Genomics (http://www.cbioportal.org/public-portal/) was searched. Both copy number variations and gene mutation data were analyzed across cancer types, focusing on HCC. The functional impact of the mutations was assessed based on evolutionary conservation of the affected amino acid in protein homologs, and the predicted functional impact score was assessed using Mutation Assessor (http://mutationassessor.org/).

### Statistical analysis

The Pearson chi-squared test was used to compare the associations between *FGF19* copy number alterations and sorafenib efficacy. All the statistical analyses were performed using JMP software (ver. 10; SAS Institute). A *P* value of <0.05 was considered statistically significant.

## SUPPLEMENTARY MATERIALS TABLE




