# Supplementary material for: Increased FGF19 copy number is frequently detected in hepatocellular carcinoma with a complete response after sorafenib treatment
Source: Oncotarget. 2016 Jun 15;7(31):49091–8. doi: 10.18632/oncotarget.10077 (PMC5226492; doi:10.18632/oncotarget.10077)
Supplement: Supplementary file 1 [file oncotarget-07-49091-s001.pdf]

Increased *FGF19* copy number is frequently detected in hepatocellular carcinoma with a complete response after sorafenib treatment

SUPPLEMENTARY TABLE

Supplementary Table S1: Copy number alteration and mutation profile in FFPE specimens obtained from HCC patients.

See Supplementary File 1
